# Orthosis-Shaped Sandals Are as Efficacious as In-Shoe Orthoses and Better than Flat Sandals for Plantar Heel Pain: A Randomized Control Trial

**DOI:** 10.1371/journal.pone.0142789

**Published:** 2015-12-15

**Authors:** Bill Vicenzino, Thomas G. McPoil, Aoife Stephenson, Sanjoy K. Paul

**Affiliations:** 1 University of Queensland, School of Health and Rehabilitation Sciences, St Lucia, Brisbane, Queensland, Australia; 2 Regis University, Rueckert-Hartman College for Health Professions, Denver, Colorado, United States of America; 3 QIMR Berghofer Medical Research Institute, Brisbane, Herston, Queensland, Australia; Bern University of Applied Sciences, SWITZERLAND

## Abstract

**Objective:**

To investigate efficacy of a contoured sandal being marketed for plantar heel pain with comparison to a flat flip-flop and contoured in-shoe insert/orthosis.

**Method:**

150 volunteers aged 50 (SD: 12) years with plantar heel pain (>4 weeks) were enrolled after responding to advertisements and eligibility determined by telephone and at first visit. Participants were randomly allocated to receive commercially available contoured sandals (n = 49), flat flip-flops (n = 50) or over the counter, pre-fabricated full-length foot orthotics (n = 51). Primary outcomes were a 15-point Global Rating of Change scale (GROC: 1 = a very great deal worse, 15 = a very great deal better), 13 to 15 representing an improvement and the 20-item Lower Extremity Function Scale (LEFS) on which participants rate 20 common weight bearing activities and activities of daily living on a 5-point scale (0 = extreme difficulty, 4 = no difficulty). Secondary outcomes were worst level of heel pain in the preceding week, and the foot and ankle ability measure. Outcomes were collected blind to allocation. Analyses were done on an intention to treat basis with 12 weeks being the primary outcome time of interest.

**Results:**

The contoured sandal was 68% more likely to report improvement in terms of GROC compared to flat flip-flop. On the LEFS the contoured sandal was 61% more likely than flat flip-flop to report improvement. The secondary outcomes in the main reflected the primary outcomes, and there were no differences between contoured sandal and shoe insert.

**Conclusions and Relevance:**

Physicians can have confidence in supporting a patient's decision to wear contoured sandals or in-shoe orthoses as one of the first and simple strategies to manage their heel pain.

**Trial Registration:**

The Australian New Zealand Clinical Trials Registry ACTRN12612000463875

## Introduction

Plantar heel pain that is present on first step out of bed in the morning or after a prolonged period of sitting is emblematic of a clinical diagnosis of plantar fasciitis. It predominantly occurs in fifth to seventh decades of life [1–3], and is estimated to affect 1 in 10 people over a lifetime [4], cost a quarter of a billion dollars per year in the United States [5,6] and lead many to consult a physician or other health care practitioner [5, 7].

Commonly recommended non-surgical treatments include ice, oral medication, stretching, orthoses, physical therapy, night splints, extracorporeal shock wave therapy and steroid injections [8]. In-shoe orthoses, either pre-fabricated or custom made [9] are generally favoured before the use of more invasive treatments like steroid injections [8, 10]. However, the effectiveness of other common treatment approaches, including sandals shaped like orthoses, is not yet known [9].

Footwear companies are increasingly incorporating aspects of foot orthoses within readily available foot wear and directly marketing to the general public without evidence of efficacy or physician consultation. An example of this is the use of contoured slip-on sandals to manage a myriad of lower limb ailments, including plantar heel pain [11,12]. Conceivably a contoured sandal, which is quicker and easier to slip on than an orthosis in a closed in shoe, that has the properties of in-shoe foot orthosis might confer advantages such as reducing first step pain and would be better tolerated in hot environments where closed-in footwear with orthoses is uncomfortable.

The objective of this study was to evaluate the efficacy of a readily available contoured sandal in the management of plantar heel pain by comparing it to a non-contoured flat flip-flop and a contoured shoe insert. We hypothesised that the contoured sandal would be more efficacious than the non-contoured flat flip-flop, and similar to a contoured shoe insert.

## Methods

### Study design and participants

**Fig 1:**
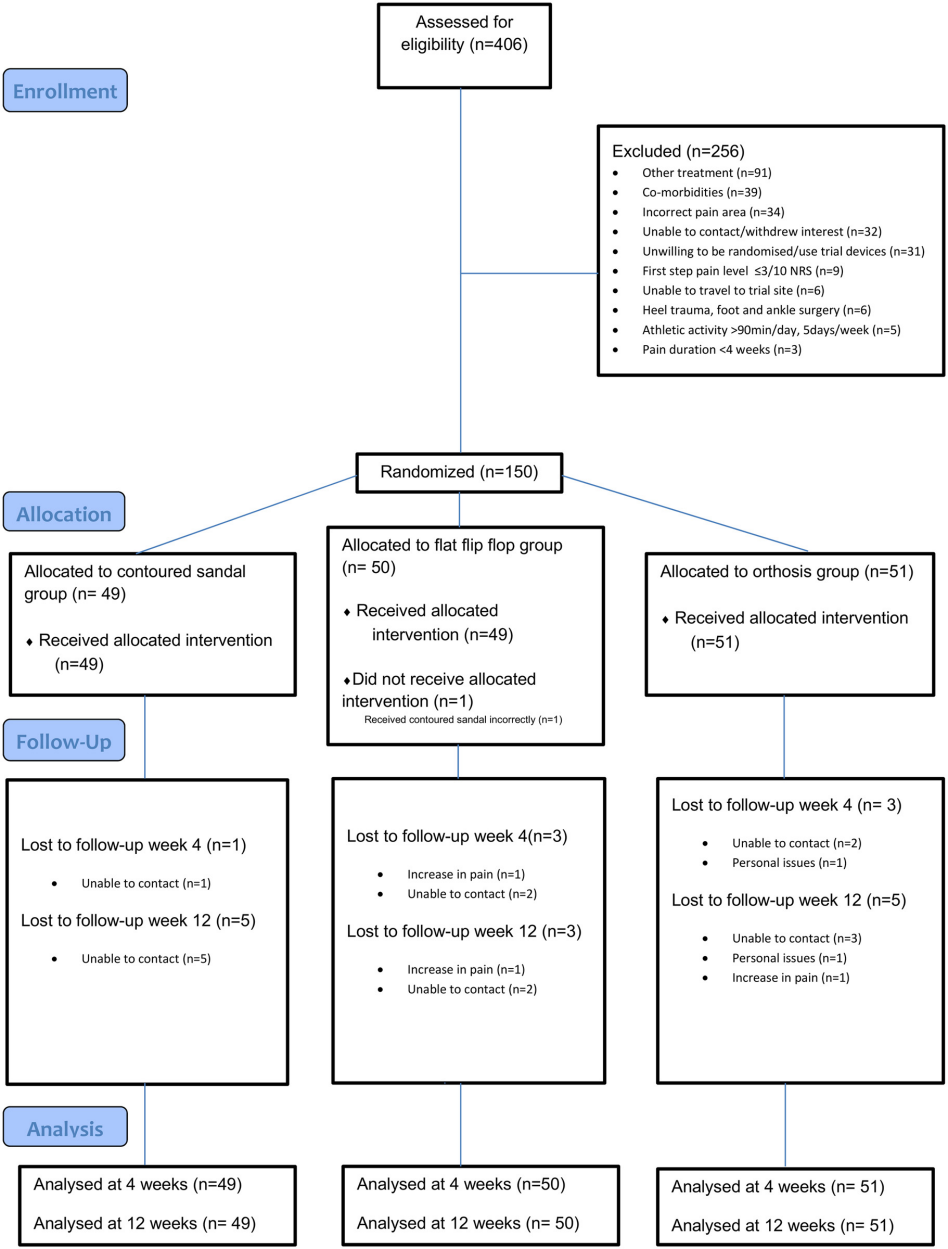
Flow of Participants through the study.

A randomized clinical trial evaluated the outcome after wearing contoured sandals compared to wearing flat flip-flops or contoured shoe inserts (over-the-counter, pre-fabricated full-length foot orthoses) over 12 weeks in patients with heel pain. Participants were followed at 4, 8 and 12 weeks (as per Fig 1) by an investigator blind to allocation. The trial was conducted across 2 Australian centres: The University of Queensland and The University of Melbourne. The University of Queensland’s Medical Research Ethics Committee approved the trial and all participants provided informed written consent. The Trial Registration Number is ACTRN12612000463875.

Adults aged 18 years or older with insidious onset, non-traumatic plantar heel pain of longer than 4 weeks duration, who responded to public advertisements between June 2012 and June 2013, were invited to participate. Inclusion criteria were: plantar heel pain of at least 3/10 on pain numerical rating scale (0 = no pain, 10 = worst pain imaginable) that was worse on first steps in the morning, willingness to wear a sandal, flip-flop or contoured shoe insert for 12 weeks; own a shoe that was adequate to fit an orthosis; and an adequate competency in written and spoken English.

Exclusion criteria were: treatment for plantar fasciitis (within past month), corticosteroid injection during preceding 6 months; pregnancy; foot pathologies (including diabetes, gout, amputation, nerve impingement, tumours, stress fracture, autoimmune disease, pitting oedema, previous plantar fascia surgery); participation in more than 90 minutes per day of athletic activity 5 or more days per week (limiting treatment of heel pain of an individual with high levels of activity was considered inappropriate); and current use of orthosis or contoured sandal. Eligibility was determined by telephone interview and confirmed on first visit to the centre, after which informed consent was obtained and baseline testing performed by an assessor blinded to allocation.

### Interventions

**Fig 2:**
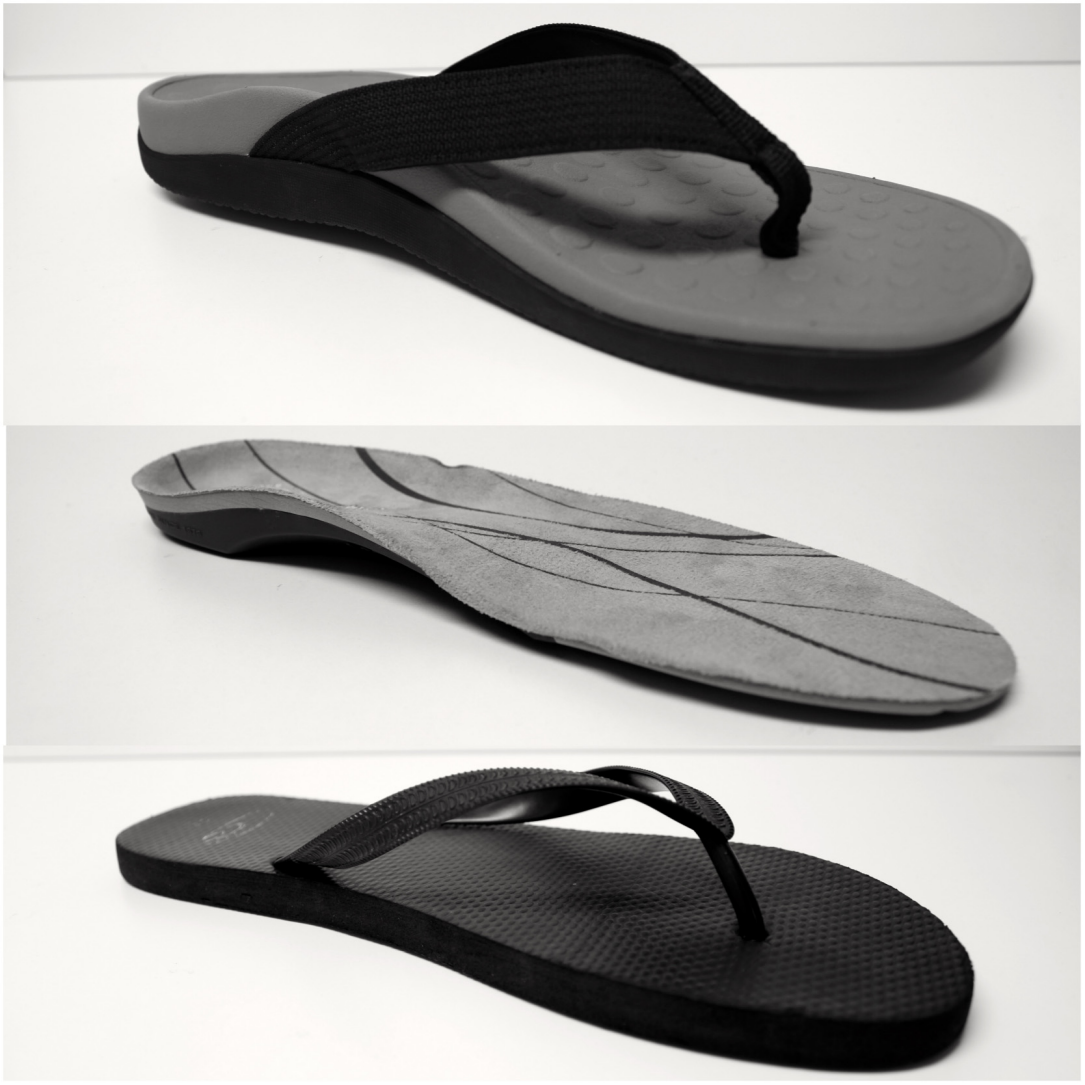
Contoured sandal, orthotic and the flat flip-flop intervention.

Participants were allocated a contoured sandal, flat flip flop or contoured shoe insert (Fig 2), which were to be worn during waking hours of the 12-week study period. The contoured sandal was a commercially available one (Vionics International, San Fransisco, USA) that has a design that approximates that of the foot-bed of a prefabricated in-shoe foot orthosis. The contoured shoe insert was a commercially available, over the counter, pre-fabricated full-length foot orthotic device (Vionics International, San Fransisco, USA). The flat flip-flop was a commercially available flat thong.

Apart from appropriate size, the key fitting guideline was that the device must be comfortable on wearing. Upon fitting, participants were asked to rate the comfort of the device on an 11 point comfort rating scale where 0 was most comfortable and 10 most uncomfortable [13]. Participants were advised to contact the investigators should any discomfort occur during the trial.

### Randomization and masking

Participants were randomly allocated to an intervention (contoured sandal, flat flip-flop, contoured shoe insert) by concealed allocation using a computer generated permuted block schedule developed by Queensland Clinical Trials and Biostatistics Centre. A research assistant other than the one taking outcome measures at baseline and during follow up administered this randomized allocation and provided the participant with their allocated device.

The research assistant who performed the outcome measures was blinded to group allocation. By the very nature of the devices, the participant and research assistant providing the devices were not blinded.

### Outcome measures

Primary outcomes were the participants’ self-reported level of improvement on a 15-point Global Rating of Change (GROC) scale (1 = a very great deal worse, 8 = no change, 15 = a very great deal better) [14] and the 20-item Lower Extremity Function Scale (LEFS) [15]. The GROC scale was dichotomised so that quite a bit better, a great deal better or a very great deal better represented a beneficial improvement/outcome, and moderately better or less on the scale (i.e., <13) represented no benefit. The LEFS seeks participant ratings on a 5-point scale (0 = extreme difficulty, 4 = no difficulty) across 20 common weight bearing activities and activities of daily living, such that a total score of 0 indicates maximum difficulty and 80 indicates no difficulty with these activities [15]. The minimal detectable change and minimal clinical important difference are both 9 points [15].

Secondary outcomes were the 11-point pain numerical rating scale (NRS; 0 = no pain, 10 = worst pain imaginable) of worst level of heel pain in the preceding week, and the 29-item foot and ankle ability measure [16]. The foot and ankle ability measure is similar to the LEFS in that it has 21-items of activities of daily living, but it also explores higher order sport activities in a further 8-items [16]. The scores are represented as a percentage for both the activities of daily living and sports subscales, with minimal detectable change of 5·7 and 12·3 respectively, and minimal clinical important difference of 8 and 9 respectively [16].

We also measured foot posture at baseline and 12 weeks. There is some conjecture as to the relationship between posture and plantar heel pain [17] and some evidence that foot orthoses control the amount of foot pronation [18]. We used the Foot Posture Index as a means of describing the foot posture in our participants with a view to evaluating if there was any change in foot posture with wearing the devices. The Foot Posture Index is a scale consisting of standardised ratings of visual observation of the foot [19]. The summation of the ratings is used to score the foot as normal (0 to 5), pronated (6 to 9)/supinated (-1 to -4) or highly pronated (10+)/supinated (-5 to -12).

A research assistant who was blinded to group allocation collected data at baseline, 4, 8 and 12 weeks.

### Statistical analysis

The primary hypothesis was that after 12-weeks of wearing the devices, the contoured sandal would outperform the flat flip-flops, but not the contoured shoe inserts. A total sample size of 150 participants was estimated to detect a 30% advantage on the dichotomised GROC scale for the contoured sandal over the flat flip-flop, assuming a 25% success rate in the latter, with 90% power, 5% type 1 error (one sided) using an online sample size calculator (http://www.stat.ubc.ca/~rollin/stats/ssize/b2.html), and approximately a 10% drop out.

All analyses were conducted on an intention-to-treat basis. The descriptive statistics were presented by number (%), mean (SD) or median (inter quartile range, IQR). Any missing values at week 12 measurements were replaced by the observed measurements at week 8.

To compare the proportions for categorical data in flat flip flop and orthosis groups, with sandal group as reference, logistic regression models were used. The odds ratios along with the 95% confidence intervals (CI) of the odds ratios were obtained, with the robust estimation of standard errors. Analyses were adjusted for baseline measure, except for the GROC measure. Numbers needed to treat and 95% CI were also calculated.

To compare continuous outcome variables, two approaches were used, first as continuous variables and second as categorical variables defined by the minimal clinical important difference at an individual participant level. The non-parametric quartile regression method was used for the continuous data, with the baseline measure for individual outcome variables as the adjustment factor. The regression coefficient estimates and their 95% confidence interval were presented. The quartile regression method was used to draw inference at the median levels of the outcome measures, due to the high skewness in the outcome variables. The categorical data was evaluated with logistic regression models as above. Analyses were conducted using STATA 12 (Texas, USA).

## Results

A total of 150 participants with plantar heel pain were enrolled and followed up between June 1 2012 and June 28 2013, 34 participants at the research site in Melbourne and 116 at the Queensland site. The random allocation of devices resulted in 49 participants (33%) receiving a contoured sandal (40 participants (34%) in Queensland and 9 participants (27%) in Melbourne), 50 (33%) receiving a flat flip-flop (37 participants (32%) in Queensland and 13 participants (38%) in Melbourne) and 51 (34%) receiving a contoured shoe insert (39 participants (34%) in Queensland and 12 participants (35%) in Melbourne).

Fig 1 summarizes recruitment, participation, and attrition. The most common reasons for exclusion of participants with suspected heel pain were recent other treatment for heel pain (36%), co-morbidities (15%), incorrect pain area (13%), unable to contact or withdrew interest (13%), and unwilling to be randomized and/or use trial devices (12%). First step pain levels too low (≤3/10 NRS), unable to travel to the research site, heel trauma, foot and ankle surgery, high athletic activity levels (>90min/day, 5days/week), and short pain duration (<4 weeks) made up the remaining 11% of excluded participants. One participant in the flat flip-flop group received a contoured sandal incorrectly. Intention to treat analysis was performed.

The trial was completed in October 2013, with 137 participants (91%) submitting primary outcomes at 12 weeks. 13 participants (9%) were lost to follow up (unable to contact n = 10, personal issues n = 1, increase in pain n = 2). Patients were deemed to have been lost to follow-up if they did not provide GROC scores. Patients who discontinued treatment had the opportunity to provide follow-up data. At 4 weeks, 143 participants (95%) completed primary outcome measures. 7 participants (5%) were lost to follow up (unable to contact n = 5, personal issues n = 1, increase in pain n = 1).

The characteristics of the study participants are presented in Table 1. The median duration of plantar heel pain symptoms in the contoured sandal group, flat flip-flop and contoured shoe insert were 24, 22 and 24 weeks respectively (Table 1). The median (IQR) of the average and worst pain rates over the past week, determined by numerical rating scales, in the contoured sandal group were, average: 4 (IQR 2–5) and worst: 7 (IQR 5–8); flat flip-flop group, average: 4 (IQR 2–6) and worst: 7 (IQR 4–8); contoured shoe insert group, average: 4 (IQR 3–5) and worst: 7 (IQR 4–8).

**Table 1:** Baseline characteristics of study subjects. (@ n(%), # mean (SD), * median (IQR), ^ 11 point comfort rating scale where 0 was most comfortable and 10 most uncomfortable)

	Contoured sandal	Flat flip-flop	Shoe insert
N(%)	49 (33%)	50 (33%)	51 (34%)
Numbers at site:			
Queensland (%/site)	40 (34%)	37 (32%)	39 (34%)
Victoria (%/site)	9 (27%)	13 (38%)	12 (35%)
Female@	32 (65%)	38 (76%)	32 (63%)
Age (years)#	52 (11)	50 (12)	50 (13)
Duration weeks*	24 (12, 56)	22 (10, 40)	24 (12, 52)
Average Pain in past week*	4 (2, 5)	4 (2, 6)	4 (3, 5)
Worst Pain in past week*	7 (5, 8)	7 (4, 8)	7 (4, 8)
Other lower limb pain@	15 (31%)	7 (14%)	15 (29%)
Foot Posture Index#	3·9 (2·85)	2·88 (2·85)	2·72 (2·33)
Comfort of device#,^	2·3 (1·9)	2·3 (2·3)	2·1 (1·8)

Other lower limb pain was reported by 15 participants (31%) in the contoured sandal group, 7 (14%) in the flat flip-flop group and 15 (29%) in the contoured shoe insert group. The Foot Posture Index scores indicated normal foot posture (Table 1). After fitting the devices, a mean (SD) comfort score of 2·3 (1·9) was reported for those in the contoured sandal group, 2·3 (2·3) for the flat flip-flop group and 2·1 (1·8) for the contoured shoe insert group.

### Primary outcomes

As measured on both the GROC and LEFS scales at the 12-week endpoint of the study, the contoured sandals provided greater benefit than the flat flip-flops but no difference compared to contoured shoe inserts. The proportion of patients who reported a beneficial improvement on GROC scores were similar between contoured sandal and contoured shoe insert groups, however significantly higher compared to those in the flat flip flop group (61% and 59% versus 34%; Table 2). Patients in the flat flip-flop group were 68% less likely to report a beneficial improvement compared to those in the contoured sandal group (95CI of OR: 0·14, 0·77; p = 0·01). No significant difference was observed between the contoured shoe insert group and the contoured sandal group (95CI of OR: 0·38, 2·09; p = 0·79).

**Table 2:** Basic statistics on global rating of change (GROC, median (IQR)), categories GROC (n, %), and the odds ratios (95% CI) for individual GROC categories for flat flip-flop and shoe insert groups, with the contoured sandal group as reference. (* p < 0·05)

	Contoured sandal	Flat flip-flop	Shoe insert
GROC week 4	11 (8, 13)	8 (8, 10)	10 (8, 13)
GROC week 8	12 (8, 14)	8 (8, 12)	12 (8, 13)
GROC week 12	13 (8, 14)	9 (8, 14)	13 (11, 14)
At week 4 [Improved ≥ quite a bit better]	14 (29%)	5 (10%)	14 (27%)
Odds Ratio (95% CI)	Reference	0·28 (0·09, 0·85)*	0·95 (0·40, 2·27)
NNT (95% CI)	Reference	-5 (-3, -34)*	-89 (-5, 6)
At week 12 [Improved ≥ quite a bit better]	27 (61%)	16 (34%)	27 (59%)
Odds Ratio (95% CI)	Reference	0·32 (0·14, 0·77)*	0·89 (0·38, 2·09)
NNT (95% CI)	Reference	-4 (-2, -15)*	-37 (-5, 6)

Participants in the contoured sandal group had a clinically meaningful 10-unit increase in LEFS score at week 12 from baseline, but neither was statistically different to the changes from baseline observed in the flat flip-flop and contoured shoe insert groups (Table 3). However, when participants were categorised on the basis of the minimal clinical important difference, those in the contoured sandal group were 61% more likely to report a change in LEFS above the minimal clinically important difference of 9 units at week 12, compared to the flat flip-flop group (95CI of OR: 0·17, 0·92; p = 0·01; Table 3). No significant difference was observed between contoured sandal and contoured shoe insert group.

**Table 3:** Basic statistics on lower extremity function scale (LEFS, median (IQR)), quantile regression coefficients (95% CI), categories of LEFS (n, %) based on the minimally clinical important difference (9 points), and the odds ratios (95% CI) for individual LEFS change categories. The regression coefficients and the odds ratios are for flat flip-flop and shoe insert, with contoured sandal as reference. (* p < 0·05)

	Contoured sandal	Flat flip-flop	Shoe insert
Baseline	60 (51, 68)	60·5 (52, 67)	58 (42, 67)
Week 4	67 (58, 72)	63 (55, 73)	65 (53·5, 71·5)
Week 8	64·5 (54, 73)	65·5 (56, 73)	64 (56, 72)
Week 12	70 (62, 76)	68 (57·5, 75·5)	71·5 (58, 78)
Change at week 12 from baseline	10 (1, 18)	6 (0, 12)	8·5 (1, 17)
Regression coefficients (effects)			
At week 4	Reference	-3·3 (-8·33, 1·7)	-0·7 (-5·6, 4·3)
At week 12	Reference	-2·7 (-8·7, 3·2)	-0·2 (-6·2, 5·8)
LEFS Change Category at week 4			
≥ 9	19 (40)	10 (22)	14 (29)
Odds Ratio (95% CI)	Reference	0·44 (0·17, 1·09)	0·63 (0·27, 1·47)
LEFS Change Category at week 12			
≥ 9	25 (57)	16(34)	24(52)
Odds Ratio (95% CI)	Reference	0·39 (0·17, 0·92)*	0·83 (0·36, 1·91)

### Secondary outcomes

The improvement in worst pain over the previous week was 1·88 (/10) units better in the contoured sandal group than the flat flip-flop group (95 CI: 0·36, 3·39, p = 0·02). No difference was observed between contoured sandal and contoured shoe insert group (S1 Table). There were no significant changes in the foot and ankle ability measure activities of daily living subscale and sport subscale at week 12. Pain and foot and ankle ability measure data are presented in S2 and S3 Tables.

The foot posture at 12 weeks as categorised by the Foot Posture Index was normal for each of the interventions: contoured sandal (mean 3·6 (SD: 2·72)), flat flip-flop (3·02 (2·40)) and contoured shoe insert (2·49 (2·23)).

Adverse events in the form of increased heel pain levels were reported by participants during the trial and occurred in both the flat flip-flop (n = 1) and contoured shoe insert (n = 1) groups. These resulted in the participants withdrawing from the study.

## Discussion

Wearing contoured sandals provides a similar beneficial effect to that of a contoured shoe insert and a superior effect to that of a flat flip-flop over a 3 month period. For every 4 patients with plantar heel pain wearing a contoured sandal, one more would be expected to be quite a bit better, a great deal better or a very great deal better than if they wore a flat flip-flop. The contoured shoe insert used in this study is similar to prefabricated in-shoe foot orthoses that have been shown to be of benefit over 3 months [9]. This supports a recommendation that the consulting physician can confidently recommend either a contoured sandal or contoured shoe insert as one of the first strategies in managing plantar heel pain. A self-evident advantage of a slip on contoured sandal over an orthosis in a closed shoe is that the patient can quickly don the sandals first thing in the morning or after prolonged sitting and avoid experiencing pain.

That the contoured sandals performed better than the flat flip-flops and similarly to contoured shoe inserts provides further support for the use of footwear with a contoured foot bed. Contoured footwear devices might exert their effect in a number of ways, most likely in a similar manner as in-shoe foot orthoses. A commonly promulgated mechanism is that the contoured device reduces rear-foot pronation [18] though the relationship between plantar heel pain and abnormal pronation is undecided [1]. Our cohort demonstrated normal foot posture as rated by the Foot Posture Index, both at baseline and at the end of the study, indicating that a pronated resting foot posture is not a feature of plantar heel pain in the studied participants and that it was not altered by wearing contoured sandals or contoured shoe inserts. Recently we demonstrated on a radiographic and anthropometric study that the contoured device raised the mid foot arch height more so than did a flat flip-flop and to a similar amount as a contoured shoe insert (orthosis) [20]. Our data might reflect that there is a temporary effect on posture by the devices but that it does not remain so after a period of wearing. Another proposition is that in-shoe foot orthoses fill the space between the foot and footwear and thus provide a greater degree of plantar surface contact between the shoe and the foot. The increased contact area might distribute stress and or provide sensory input across the plantar soft tissues including the plantar fascia and thereby help relieve pain [21, 22].

There are some limitations of this study that ought to be kept in mind when interpreting the results. We did not ask if first step pain had changed, but it would be reasonable to assume that the GROC score would be representative of any improvement or worsening of first step pain. Compliance to device wear was not evaluated and so we were unable to ascertain if compliance influenced the outcomes, but we did use an intention to treat analytic approach that by definition indicates our results apply to the decision to wear the device as much as if it was worn, how much or not at all. Another caveat on the interpretation of the results is that we were unable to blind the patients to the device they were allocated, so a placebo mechanism might explain the beneficial effects of the contoured sandal and contoured shoe insert over the flip-flop. However, a previous randomized placebo controlled trial reported orthoses, which were similar to the ones used in our trial, produced beneficial effects that were superior to placebo effects over 3 months [23]. That the contoured sandals produced similar outcomes to that of the contoured shoe insert studied herein implies that the contoured sandals produce effects beyond that of placebo.

## Supporting Information

S1 Table Basic statistics for pain in the previous week and median difference. (DOCX)

S2 Table Basic statistics on foot ankle ability measure—activities of daily living subscale. (DOCX)

S3 Table Basic statistics on foot ankle ability measure—sport subscale (FAAM-Sport, median (IQR)), quantile regression coefficients (95% CI), categories of FAAM-Sport (n, %) based on the minimally clinical important difference (9 points), and the odds ratios (95% CI) for individual FAAM-Sport change categories. The regression coefficients and the odds ratios are for flat flip-flop and shoe insert, with contoured sandal group as reference.
(DOCX)

S1 File CONSORT-2010-Checklist-MS-Word. (DOC)

S2 File Protocol_OFFPHtrial. (PDF)

S3 File Ethics Approval OFFPH_20120207. (PDF)
