# Supplementary material for: Orthosis-Shaped Sandals Are as Efficacious as In-Shoe Orthoses and Better than Flat Sandals for Plantar Heel Pain: A Randomized Control Trial
Source: PLoS One. 2015 Dec 15;10(12):e0142789. doi: 10.1371/journal.pone.0142789 (PMC4686010; doi:10.1371/journal.pone.0142789)
Supplement: S1 Table — (DOCX) [file pone.0142789.s001.docx]

**S1 Table : Basic statistics (median (IQR)) for pain in the previous week and median difference (95% CI). The regression coefficients are for flat flip-flop and shoe insert, with contoured sandal as reference.**

|  | Contoured sandal | Flat flip flop | Shoe insert |
| --- | --- | --- | --- |
| Baseline | 7 (5, 8) | 7 (4, 8) | 7 (4, 8) |
| Week 4 | 4·5 (3·0, 6·5) | 4 (2, 7) | 4 (3, 7) |
| Week 8 | 4 (2, 7) | 3 (2, 6) | 3 (2, 7) |
| Week 12 | 3 (1, 5) | 3 (1, 6) | 2 (1, 5) |
| Change at week 12 from baseline | -4 (-6, -1) | - 2 (-5, -1) | -3 (-6, -1) |
| Median difference (effects) | | | |
| At week 4 | Reference | 0·14 (-1·11, 1·39) | -0·29 (-1·54, 0·97) |
| At week 12 | Reference | 1·88 (0·36, 3·39)! | 0·25 (-1·25, 1·75) |

! p <0·05
