# Supplementary material for: Orthosis-Shaped Sandals Are as Efficacious as In-Shoe Orthoses and Better than Flat Sandals for Plantar Heel Pain: A Randomized Control Trial
Source: PLoS One. 2015 Dec 15;10(12):e0142789. doi: 10.1371/journal.pone.0142789 (PMC4686010; doi:10.1371/journal.pone.0142789)
Supplement: S2 Table — (DOCX) [file pone.0142789.s002.docx]

**S2 Table: Basic statistics on foot ankle ability measure – activities of daily living subscale (FAAM-ADL, median (IQR)), quantile regression coefficients (95% CI), categories of FAAM-ADL (n, %) based on the minimally clinical important difference (8 points), and the odds ratios (95% CI) for individual FAAM-ADL change categories. The regression coefficients and the odds ratios are for flat flip-flop and shoe insert, with contoured sandal as reference.**

|  | Contoured sandal | Flat flip flop | Shoe insert |
| --- | --- | --- | --- |
| Baseline | 77 (67, 85) | 76 (70, 86) | 71 (56, 86) |
| Week 4 | 84 (72, 93) | 82 (71, 90) | 80 (69, 92) |
| Week 8 | 82 (73, 95) | 84 (71, 94) | 83 (69, 94) |
| Week 12 | 89 (80, 96) | 88 (75, 96) | 93 (72, 98) |
| Change at week 12 from baseline | 10 (2, 21) | 6 (-4, 18) | 11 (3, 20) |
| Regression coefficients (effects) | | | |
| At week 4 | Reference | -2·8 (-9·2, 3·6) | 0·3 (-6·1, 6·7) |
| At week 12 | Reference | -4·3 (-12·0, 3·5) | -0·2 (-7·6, 8·0) |
| ADL Change Category 1 at week 4 | | | |
| ≥8 | 20 (45) | 12 (27) | 19 (40) |
| Odds Ratio (95% CI) | Reference | 0·44 (0·17, 1·06) | 0·81 (0·35, 1·88) |
| ADL Change Category 1 at week 12 | | | |
| ≥ 8 | 25 (58) | 22 (51) | 27 (60) |
| Odds Ratio (95% CI) | Reference | 0·75 (0·32, 1·77) | 1·08 (0·46, 2·54) |
