# Supplementary material for: Orthosis-Shaped Sandals Are as Efficacious as In-Shoe Orthoses and Better than Flat Sandals for Plantar Heel Pain: A Randomized Control Trial
Source: PLoS One. 2015 Dec 15;10(12):e0142789. doi: 10.1371/journal.pone.0142789 (PMC4686010; doi:10.1371/journal.pone.0142789)
Supplement: S3 Table — The regression coefficients and the odds ratios are for flat flip-flop and shoe insert, with contoured sandal group as reference. (DOCX) [file pone.0142789.s003.docx]

**S3 Table: Basic statistics on foot ankle ability measure - sport subscale (FAAM-Sport, median (IQR)), quantile regression coefficients (95% CI), categories of FAAM-Sport (n, %) based on the minimally clinical important difference (9 points), and the odds ratios (95% CI) for individual FAAM-Sport change categories. The regression coefficients and the odds ratios are for flat flip-flop and shoe insert, with contoured sandal group as reference.**

|  | Contoured sandal | Flat flip flop | Shoe insert |
| --- | --- | --- | --- |
| Baseline | 66 (50, 78) | 66 (50, 82) | 56 (38, 72) |
| Week 4 | 73 (58, 91) | 72 (53, 82) | 72 (50, 86) |
| Week 8 | 75 (64, 91) | 75 (60, 91) | 73 (55, 93) |
| Week 12 | 84 (71, 100) | 75 (59, 91) | 78 (59, 98) |
| Change at week 12 from baseline | 16 (3, 28) | 9 (-3, 28) | 16 (3, 34) |
| Regression coefficients (effects) | | | |
| At week 4 | Reference | -5·29 (-14·30, 3·72) | -2·28 (-11·40, 6·85) |
| At week 12 | Reference | -9·66 (-20·88, 1·56) | -3·41 (-14·70, 7·88) |
| Sport Change Category 1 at week 4 | | | |
| ≥9 | 22 (50) | 13 (29) | 20 (43) |
| Odds Ratio (95% CI) | Reference | 0·41 (0·17, 0·98)! | 0·77 (0·33, 1·77) |
| Sport Change Category 1 at week 12 | | | |
| ≥ 9 | 28 (65) | 22 (54) | 27(61) |
| Odds Ratio (95% CI) | Reference | 0·62 (0·26, 1·50) | 0·85 (0·35, 2·04) |

! p < 0·05
