# Supplementary material for: Orthosis-Shaped Sandals Are as Efficacious as In-Shoe Orthoses and Better than Flat Sandals for Plantar Heel Pain: A Randomized Control Trial
Source: PLoS One. 2015 Dec 15;10(12):e0142789. doi: 10.1371/journal.pone.0142789 (PMC4686010; doi:10.1371/journal.pone.0142789)
Supplement: S2 File — (PDF) [file pone.0142789.s005.pdf]

Orthoses or Flip-Flops for Pain in the Heel (OFFPH) trial protocol.

1. **Objectives:**
   The primary objective of this study is to evaluate the efficacy of a contoured sandal (SANDAL) compared to a: (a) flat thong (THONG) or (b) shoe insert (ORTHOSIS).

2. **Hypotheses to be tested:**
   a) SANDAL will result in a better outcome than a THONG.
   b) SANDAL will result in a similar outcome to an ORTHOSIS.

3. **Methods**
   - Random allocation to either treatment (SANDAL, THONG, ORTHOSIS)
   - Scheduled drawn up by QCTC
   - Concealed by sealed envelope
   - Two centres: UQ and University of Melbourne
   - Data collected blind to allocation
   - Data collected at baseline, 4, 8 and 12 weeks – primarily interested in the 12 week outcomes, and possibly the short term 4 week outcome. I have included all time points so that if one way of treating missing data is to carry forward the last measure, then there will be some data to use.
   - Devices provided to the participants by a non health care professional so as to imitate the situation whereby these devices are available in a non-health care profession (retail) setting

4. **Primary outcomes:**
   a) 15 point Global Rating Of Change scale (GROC4, GROC8, GROC12)(1,2): participants were asked to *'Please rate the overall progress of your heel pain'* on a 15 point scale, where 1 is the worse case scenario and 15 is the best case scenario (variable names:GROC4,GROC12). The score is dichotomised to 'improved' versus 'not improved': whereby improved is 'quite a bit better (13)', 'a great deal better (14)' and 'a very great deal better (15)', which is GROC4Cat1 and GROC12Cat1 (i.e., at 4 and 12 weeks). We have also dichotomised at 'Moderately better (12)' [GROC4Cat2, GROC12Cat2] and at 'A tiny bit better (9)' [GROC4Cat3, GROC12Cat3] – in order to evaluate the effect of the cut point on the outcome.

Please rate the overall progress of your heel pain:

a. ***A very great deal better (15)***
b. ***A great deal better (14)***
c. ***Quite a bit better (13)***
d. **Moderately better (12)**
e. **Somewhat better (11)**
f. **A little bit better (10)**
g. **A tiny bit better (9)**
h. **Same – No Change (8)**
i. **A tiny bit worse (7)**
j. **A little bit worse (6)**
k. **Somewhat worse (5)**
l. **Moderately worse (4)**
m. **Quite a bit worse (3)**
n. **A great deal worse (2)**
o. **A very great deal worse (1)**

b) Lower Extremity Function Scale (LEFS0, LEFS4, LEFS8,LEFS12)(3,4): participants rate 20 tasks on a 5 point scale where 0 is unable to do/extremely difficult and 4 is no difficulty. The score is out of 80 and 9 has been calculated as a minimum level of detectable change.

the minimal detectable change is 9 scale points (90% CI), and the minimal clinically important difference is 9 scale points (90% CI){Binkley:1999uq}

Primary outcome time points of interest

c) Baseline, 4 and 12 weeks (note1: GROC has no baseline; note2: measured at 8 weeks as well but not included in the data base)

5. Secondary outcomes

a) Foot and Ankle Ability Measure (FAAM)(5): This is a similar questionnaire to LEFS with a 5 point scale but 30 questions, which are divided into two parts, one for normal day to day activities (ADL) and the other is for higher level sport activities (SPORT). There will be 2 FAAM scores: (i) FAAMADL0, 4, 8 and 12 and (ii) FAAMSport0, 4, 8 and 12. This scale allows for non-answers. It is scored by dividing the sum of the scores with the total score possible for the number of questions answered (i.e., if only 10 questions answered, then the denominator becomes 40 and if the patient ratings on these 10 questions sums to 20, the score is 50%)

The minimal detectable change based on a 95% confidence interval was ±5.7 and ±−12.3 points for the ADL and Sports subscales, respectively. The minimal clinically important differences were 8 and 9 points for the ADL and Sports subscales, respectively.{Martin:2005vr}

b) Average Pain Numerical Rating Scale over past week (avPAIN0, 4, 8 and 12): participants rate their average pain experience on a 0 to 10 scale with 0 being no pain and 10 being worst pain imaginable over the previous week. These were measured

c) Worst Pain Numerical Rating Scale over past week (weekPAIN0, 4, 8 and 12) and over past day (dayPAIN0, 4, 8 and 12): this is the same as the preceding, but patient is rating their worst level of pain in the past week and day respectively.

6. Data management and statistical analysis:

- Primarily interested in the GROC and to a lesser extent LEFS.
- Could we use the GROC categorisation into dichotomised (either GROC12Cat1 or 2) as a grouping variable, so that we can see how different the LEFS is across the cohort (and if it differs according to Treatment and Time factors)?
- Sample size estimate of 44 per group was based on aiming to detect a 30% advantage on the GROC scale (dichotomised) for the Sandal over the thong (assuming a 25% success rate in the thong), alpha 95% and power 90%. An additional 6 participants per group were factored in to accommodate for dropouts (12%). This is similar to the sample size calculated for the LEFS based on a difference between groups in the order of the MDC of 9 and a SD 1.5 this difference (assuming alpha 95% and power 90%). <<<<this sample size is similar to others examining orthoses(6)>>>
- Intention to treat: all analyses to be conducted on an ITT basis

- Missing data managed – how? Have provided all time points in case using carry last data point forward method (which I think might be an ok thing to do in this context as all measures occurred within 3 months and likely trajectory is to continue in a similar path)
- Baseline characteristics and demographics table required
- Consort participant flow chart required (Figure)
- P value = 0.05
- Categorical Data to be represented as:
    i. Frequency count, denominator and proportion (%) for categorical data
    ii. Absolute Risk, Relative Risk, NNT and 95% confidence intervals for comparisons of interest (SANDAL v THONG, SANDAL v ORTHOSIS) categorical data
- Ratio (Continuous) Data to be represented as:
    i. Group mean and SD for descriptives
    ii. Mean differences and 95% confidence intervals for the comparisons: SANDAL v THONG and SANDAL v ORTHOSIS.
    iii. Standardised mean differences and 95% confidence intervals
    iv. Interaction plots to be plotted for any interaction effects

7. References:

1. Kamper SJ, Maher CG, Mackay G. Global rating of change scales: a review of strengths and weaknesses and considerations for design. J Man Manip Ther. 2009;17(3):163–70.

2. Kamper S. Global Rating of Change scales. Aust J Physiother. 2009;55(4):289.

3. Binkley JM, Stratford PW, Lott SA, Riddle DL. The Lower Extremity Functional Scale (LEFS): scale development, measurement properties, and clinical application. North American Orthopaedic Rehabilitation Research Network. Phys Ther. 1999 Apr 1;79(4):371–83.

4. Binkley. Lower Extremity Function Scale. scale pdf. 2002 Oct 4;:1.

5. Martin RL, Irrgang JJ, Burdett RG, Conti SF, Van Swearingen JM. Evidence of validity for the Foot and Ankle Ability Measure (FAAM). Foot Ankle Int. 2005 Nov 1;26(11):968–83.

6. Landorf KB, Keenan A-M, Herbert RD. Effectiveness of foot orthoses to treat plantar fasciitis: a randomized trial. Arch Intern Med. 2006 Jun 26;166(12):1305–10.
