# Supplementary material for: Orthosis-Shaped Sandals Are as Efficacious as In-Shoe Orthoses and Better than Flat Sandals for Plantar Heel Pain: A Randomized Control Trial
Source: PLoS One. 2015 Dec 15;10(12):e0142789. doi: 10.1371/journal.pone.0142789 (PMC4686010; doi:10.1371/journal.pone.0142789)
Supplement: S3 File — (PDF) [file pone.0142789.s006.pdf]

CLEARANCE Nº (office use only)

THE UNIVERSITY OF QUEENSLAND

Application Form for Ethical Clearance for Research Involving Human Participants

For review by: **Medical Research Ethics Committee (MREC)**
Behavioural & Social Sciences Ethical Review Committee (BSSERC)
For Staff and Student Research
Refer to last page for website and other information, including mailing address

Please tick boxes:

MREC	X	BSSERC	

Full Review	X	Expedited Review	

ALL QUESTIONS MUST BE ANSWERED

- **minimum 12 point font**
- **define any acronyms and abbreviations used**

Project Title:
**Orthoses or Flip-Flops for Pain in the Heel (OFFPH study): a randomised clinical trial.**

**Principal Investigator:**	Professor Bill Vicenzino
**Staff Nº/Student Nº:** (cross out if not relevant)	1037774

**Co-Investigator/s:**	Professor Thomas McPoil (Regis University, Denver, CO, USA, Honorary Prof, SHRS, UQ)<br>Professor Paul Mintken (University of Colorado, Denver, CO, USA)
**Project Co-ordinator (or authorised contact)**	Professor Bill Vicenzino

**Supervisor/s:** (if applicable)	N/a

**Schools/Departments:**	School of Health and Rehabilitation Sciences

	Telephone	Fax	Email
**Contact details of Principal Investigator**	**+61 7 3365 2781**	**+61 7 3346 7332**	**b.vicenzino@uq.edu.au**
**Contact details of Project Co-ordinator or authorised contact**	as above	as above	as above

**Degree Enrolled (if student):**	N/a
**Funding Body:**	Vasyli International, USA
**If Project Funded - What year?<br>- Reference no. if available**	2012 [Consultancy]

**Project Location:**	School of Health and Rehabilitation Sciences, UQ<br>Regis University, Denver, Colorado, USA<br>University of Colorado, Denver, Colorado, USA	**Project Duration:**	18 months

A. Is this submission identical or very similar to a previously approved protocol? **NO** (circle)

If **YES**, please provide clearance noº **and** indicate whether identical or very similar):______________________

B. Does this submission hold other ethical clearance? **NO** (circle)

Note: Copies from other **AHEC** registered ethics committees must be attached.

C. Are you applying for Expedited Review? **NO** (circle)

Note: Please see UQ Guidelines page 10 for the conditions necessary to qualify for Expedited Review.

D. Is the project a Clinical Trial (eg, a trial of a drug, device, therapy, intervention, treatment, etc) ? [refer to end of this form dealing with "clinical trials"] **YES** (circle)

If **YES**, please specify:_This is a randomised clinical trial that is testing the clinical efficacy of an in shoe orthoses (usually fitted by a registered practitioner) and flip-flops on heel pain.

PLEASE ANSWER ALL OF THE FOLLOWING QUESTIONS:

1) Who are the participants or informants?: eg, Children, University students, or other persons.

Note: Details of inclusion/exclusion criteria including approximate **number** (provide justification), age range, and male/female ratios are required.

Participants will have pain under the heel (plantar heel pain) and be either female or male and adult.

We will recruit 150 participants, based on a 10% drop out rate and previous studies of orthoses in plantar heel pain showing significant clinical effects. These 150 participants will be divided into groups of 50, with 75 participating at UQ and 75 at a designated site in Colorado (Denver and Bolder).

Selection criteria for participants:

- Report of (insidious non-traumatic onset) plantar heel pain for at least 1 month
- First step pain in the morning of at least a 3/10 on a Pain Numerical Rating Scale
- Adequate English communication skills to allow valid treatment and outcome measurements to be performed
- Adequate shoes into which the active comparator can be fitted
- Willingness to wear a flip-flop or orthosis regularly for the 12 weeks duration of the trial
- English communication skills required to adequately understand questionnaires, treatments (orthoses) and outcome measures
- Willingness to wear a flip-flop or orthosis regularly for 12 weeks during the trial

Exclusion criteria for participants:

- History of previous physical therapy treatment of plantar fasciitis within the past month
- History or diagnosis of circulatory conditions
- Pregnancy
- Foot pathologies (including diabetes, gout, amputation, nerve impingement, tarsal tunnel syndrome, tumours, stress fracture, autoimmune disease, pitting oedema, lower extremity referred pain, previous plantar fascia surgical procedures, corticosteroid injections) within the past 12 months (for the transient conditions or treatments)
- Participation in athletic activity 5 or more days per week for more than 90 minutes per day
- Other orthopaedic or neurological impairment that prevents a symmetrical walking pattern, or an antalgic gait that was not attributed to plantar heel pain

2) Special Groups

The *National Statement* has identified certain groups with specific ethical considerations. Researchers must take special care to protect the interests of these groups if they are in any way involved in the project. Those groups include: **pregnant women and the foetus** (Ch 4.1); **children and young people** (Ch 4.2); **people in dependent or unequal relationships** (Ch 4.3); **people highly dependent on medical care** (Ch 4.4); **people with cognitive impairment, intellectual disability, or mental illness** (Ch 4.5); **people involved in illegal activities** (Ch 4.6); **Aboriginal and Torres Strait Islander peoples** (Ch 4.7); **people in other countries** (Ch 4.8); **other cultural and ethnic groups**.

In preparing your research project and application for ethical clearance, you should investigate thoroughly, through consultation with supervisors, colleagues in your school and other professional groups/organizations, how these special groups may or may not be represented in your research and if participation in this research could have a negative impact on members of any of these groups.

Note: If participation of special groups is a focus of the research, the protocol can not qualify for expedited review (unless other current HREC clearance is held and a copy provided).

2a) Aboriginal and Torres Strait Islanders Group

Specify the level of participation that Indigenous Australians will have in this research (as members of the research team, or as members of the group to be researched):

no participation	**some participation possible or likely**	focus of the research
☐	X	☐

Please explain your choice:

It is possible that some participants will identify as Aboriginal or Torres Strait Islander, but this is not the focus of the research. Selection will be based on plantar heel pain and related conditions as outlined above. No preference will be given based on indigenous or non-indigenous status. For all participants, English communication levels must be at a sufficient level to facilitate participation and outcome measurements. Note that participation of Aboriginal or Torres Strait Islander peoples is limited to some extent by the fact that 50% of participants will be recruited in the US.

If Indigenous Australians may be involved (2nd or 3rd response box above), what strategies will be used to address their needs and interests? [For guidance with this part of Q2a on indigenous and cultural issues, please refer to the NHMRC and AIATSIS codes of ethics for research with indigenous people. For further advice please contact the UQ Aboriginal and Torres Strait Islander Studies Unit.]

Please specify your strategies:

The research team does not anticipate that participation in the study will cause distress or discomfort to Aboriginal or Torres Strait Islander people. However, if unique needs are identified for people in this group, the research team will consult the NHMRC and AIATSIS codes of ethics, and/or the UQ ATSIS Unit for guidance.

2b) People in Australia belonging to other cultural or ethnic groups

Are there any ethical considerations that may arise as a result of collection from other cultural or ethnic groups in Australia? [for example, are there any particular customs, practices, or conditions which should be taken into account]: NO

If YES, please provide details:

Have you consulted anyone with knowledge to provide guidance? Who?:

2c) People in overseas countries

Does your project involve data collection in an overseas country?: YES

If YES, what ethical considerations may arise as a result of such data collection, which are different from those arising from data collection in a general Australian context? [for example, are there any particular **local** laws, customs, practices, or conditions which should be taken into account?]:

Fifty percent of participants will be recruited by Prof McPoil and Prof Mintken in the United States. No different ethical considerations have been identified for these participants, as cultural practices and assumptions are very similar in the US and Australia. Data collection undertaken in the US will be led by the American members of the research team, who will have a first hand understanding of any ethical considerations unique to local participants. The relevant Institutional Review Boards in Colorado will review the project prior to commencement.

Have you consulted anyone with knowledge to provide guidance? Who?:

The Australian and American members of the research team have devised the study collaboratively; therefore it has been deemed appropriate for both participating countries.

2d) Other Special Groups

Does your project involve any of the other special groups (listed above in the introduction to Q2)?: YES

If YES, please answer the following:

Specify the group/s:

Pregnant women, young people, other cultural and ethnic groups

What is the level of their participation:

**some participation possible or likely**	focus of the research
X	☐

What strategies will be used to address their needs and interests?

Please specify your strategies:

In all cases, participation is entirely voluntary and may be withdrawn at the discretion of the participant. Detailed information about study activities will be provided to all applicants prior to recruitment. Participants will be encouraged to direct any questions or concerns to members of the research team throughout the project duration.

**3a) Participant recruitment details: Please provide exact details of contact.**
Participants will be recruited from the general community through advertisements (local notice boards, print and radio media) and media releases. Potential participants replying to the advertisements and media release will be screened over the phone (or in some cases by email) and if deemed to be suitable followed up with a further screening examination at the research centres. If then the participant meets selection criteria they will be entered into the trial. Information will be provided both over the phone (or by email) and at the face to face screening examination, prior to the participant consenting (in writing) before being randomised into the trial.
**3b) Does recruitment include disclosure of personal information (eg, mailing list, names, contact details, etc) from another party or organisation to the researchers? If <u>YES</u>, please provide details.** **NO**<br>**Note:** disclosure of personal information from another party or organisation to the researchers, even if merely for the purpose of seeking initial expression of interest in the project, must be authorised by each individual to whom the information relates (unless it is a completely public database with unrestricted access). Eg, Clinic X must not give to the researchers a mailing list of patients who might be potential participants for the project unless those patients have previously authorised such use and disclosure of their information to non-clinic parties.

4) In <u>EVERY-DAY</u> or <u>LAY LANGUAGE</u> please provide a summary of the project – including aims and benefit: This section <u>MUST</u> be completed in <u>LAY LANGUAGE</u>.

Plantar heel pain is a common problem for many people in Australia and the US. There is some high quality evidence that in-shoe foot orthoses are helpful in resolving pain and disability associated with plantar heel pain. These orthoses often are prescribed to control excessive pronation, which is thought to be a mechanical factor contributing to overload and eventual pain of the tissues in the plantar heel region (known as planta fascia). Recently flip-flops have been designed with features similar to that of the orthoses, but without the need to be fitted within a shoe. A potential benefit of a flip-flop is that the substantial pain levels experienced on arising from bed in the morning, during the night or out of a chair might be averted if a flip-flop in the shape of an orthosis is in place on first weight bearing. The flip-flop being quicker and easier to fit than a shoe, especially in circumstances when shoes are not already on the feet prior to weight bearing. This study will employ a randomised clinical trial methodology to evaluate the differential effects of an in shoe foot orthosis, a contoured flip flop versus a standard flat flip flop in a sample of people with plantar heel pain.

The participants will be randomly allocated to either of the three possible treatments and their allocation will be concealed from the investigator who recruits them into the study. The randomisation schedule will be drawn up by an independent centre (Queensland Clinical Trial Centre). The therapist and participant, by nature of the devices being studied, cannot be blinded. The key criterion for all devices is that they are comfortable to wear. That is, no participant will wear an uncomfortable device. The participant will wear the orthoses or flip-flops for 12 weeks over which time (4, 8 and 12 weeks) they will complete some outcome measures, which indicate the effects of the devices and state of their heel pain. In addition to the paper based outcome measures all participants will have their feet measured at baseline and at the end of the trial. All outcome measures are non-invasive. The investigator who collects the outcome data will be blind to group allocation. Data will be analysed blind to allocation and on an intention to treat basis.

An opportunity presents itself by means of a similar foot bed (shape and form) being present in an in shoe foot orthosis and a flip-flop, both being manufactured by one company (Vasyli Internationl), hence a level of consistency between the direct interaction effects of the orthoses or flip-flop with the foot. Vasyli International will supply all materials (orthoses and flip-flops) as well as provide funds to cover costs associated with advertising (to recruit participants), therapist's fees (for fitting of flip flops and orthoses) and a small payment to participants to cover some of the expenses involved in attending the research labs where screening and some outcome measures will be performed (e.g. public transport, parking). The company will not have any further role in the design, planning and conduct of the study. Vasyli will have no role in any of the data analysis and its interpretation, nor of its reporting.

5) Give details of the research plan:

Note: The committee needs sufficient information to put into context the ethical considerations listed in later questions.

Note: This section should be completed in LAY LANGUAGE *as much as possible* so that it can be understood and appreciated by all Committee Members, including Lay Members.

Note: For application to the MREC – please keep response to a MAXIMUM of 2 pages.

Following consenting to be in the study and prior to being randomly allocated to a group, participants will undergo baseline measurements on a battery of measures listed following:

- Primary outcome measures
  1. 15 point Global Rating of Change Scale: participants are asked to rate if they are better, same or worse, and if better or worse by how much on a 7 point scale (hence 15 points, 7 worse, 1 same and 7 better). This is a commonly used rating of change scale in clinical trials.
  2. Lower Extremity Function Scale: Is a 20 item scale in which the participant rates the degree of difficulty (extreme to no difficulty over 0 to 4 points) on common weight bearing activities.

- Secondary outcome measures
  3. Foot and Ankle Ability Measure: Is similar to the LEFS above (#2) but has 30 items, in two main parts, one dealing with higher order sports activities. It is used in order to cover the possibility of higher activity levels in our cohort. The clinicometric properties are otherwise similar to #2.
  4. Pain Numerical Rating Scale: An 11 point scale anchored by no pain at all at 0 and worst pain imaginable at 10 will be used to rate the worst and average level of pain severity the patient experienced in the past 24 hours and past week.
  5. Patient Specific Function Scale: The participant choses a maximum of five tasks that they no longer can do unimpeded by their heel pain and scores the level of disability for each of these tasks on a 11 point numerical rating scale, where 0 is no interference to function and 10 is maximal interference with the task (i.e. no function, as in wheel chair bound or non-weight bearing on crutches)
  6. Foot Posture Index: This is a scale consisting of ratings of therapist's observation of foot posture commonly used to score foot posture as pronated, neutral or supinated.
  7. Mid-foot height barefoot and in flip-flops
  8. Mid-foot width barefoot and in flip-flops: The mid foot measures are taken in weight bearing and non weight bearing with digital calipers, a non-invasive and non-painful procedure taking under 3 minutes (both feet weight bearing and non weight bearing)

All these measures are painless, quick and easy to complete, all except 7 & 8 being commonly used in clinic.

At 4 weeks, 8 weeks and 12 weeks after baseline measurement, participants will be asked to again complete all of the measures with the exception that measures 6, 7 & 8 will only be performed at baseline and 12 weeks. The reason for these measures being taken at these two time points, is (i) there is preliminary evidence that mid foot width measures are predictive of success for some other conditions that are treated by orthoses, and (ii) orthoses (and the like shaped flip-flop) might alter foot form that might be picked up on these measures.

Data will be analysed according to current best practice including use of baseline measures as covariates where appropriate and appropriate methods to control for type I error. Numbers needed to treat (NNT) will be calculated from measure #1, through dichotomisation. This will provide health care providers with meaningful data on which to base their decisions about using orthoses or flip-flops in management of plantar heel pain.

6) Give details of the ethical considerations attached to the proposed project:

There are no significant ethical considerations identified as relating to this project. The project aims to identify a possible method for alleviating paint for people with existing plantar heel pain. There is no identified risk of worsening of any participant's condition, especially as the devices will be comfortable to wear. Involvement is entirely voluntary, and participants may withdraw from the study at any time at their own discretion.

As part of the initial and follow-up assessments, participants will be required to report on personal pain and function levels. In some cases this may require disclosure of the cause of pain, which is a potential cause of distress for participants. However, it is considered highly unlikely that participants will become distressed, as only those reporting non-traumatic onset plantar heel pain will be recruited to the study.

7a) How will informed consent be obtained from participants or informants?

Those interested in participating in the study will receive a written information pack detailing study aims, activities, requirements, selection and exclusion criteria, and projected outcomes. Contact details of researchers will be provided for the purposes of making inquiries or seeking clarifications. It will be established in the information pack that involvement in the study is entirely voluntary, and that participants may withdraw at any stage and at their own discretion.

After reading this information, those who wish to participate in the study will be asked to sign a written consent form, which will then be returned to researchers at their local study site (i.e. UQ or the designated Colorado site).

7b) "Gatekeeper" Approvals

A "gatekeeper" or "permission-giver" is a person authorised to write a Letter of Authority and Recognition from an organisation of any type involved with the research, which gives permission to the researcher for access to the population **under the "gatekeeper's" or "permission-giver's" authority**.

[For example, if you wish to conduct research in schools and the participants are the school teachers, then gatekeeper approval will need obtained from the relevant education authority (eg, Education Queensland) and the School Principals before you may approach those school teachers in recruitment.

For example, if you wish to access staff from a private organisation, then similarly, gatekeeper approval will usually be required from senior personnel or an appropriate manager who is able to grant such access to approach that organisation's staff in recruitment.]

1. Are gatekeeper approval/s required for the research?: NO

2. If **YES**, who are the gatekeeper/s and how will their approvals be sought and obtained? (if gatekeeper approval/s have already been obtained, then please attach copy)

8) Provide details of procedures for establishing confidentiality and protecting privacy of participants or

informants:

Participants will be personally identifiable during recruitment and data collection; however, only anonymous and generalised data will be used in the dissemination of study findings. Individual electronic data will be linked to consent forms by an assigned number, so that only members of the research team can link individual results to participants. (Allowing for the re-identification of participants is necessary to facilitate participant withdrawal from the study if requested.) Consent forms will be stored separately from data to maintain participant privacy. Consent forms and individual results will be accessible only to members of the research team.

9) Researchers must ensure that all data, particularly data containing personal information (ie, information that can identify the person), are secure both at the point of storage and during transit. Researchers must be aware of relevant legislation and guidelines governing privacy:- *Information Privacy Act* (Qld) 2009, *Privacy Act* (Cth) 1988, and Guidelines under S95 and S95A of the *Privacy Act* (Cth).

9a) Where will data be stored (eg, UQ office of researcher), and what measures will be taken to ensure security of data (eg, locked filing cabinets, computer hard-drive protected by password/encryption/de-identification of data, etc)?

Data will be stored in the offices of the research team, both at UQ and at the participating university facilities in Colorado. Electronic data will be located on password-protected computers, and hard copy documents will be filed and stored in the researchers' locked offices.

9b) Will data be stored on, or taken to, premises other than secure UQ premises (eg, researcher's home)?: YES

If YES, then what measures will be taken to ensure security of data at these premises?

It is possible that data will be accessed outside the secure university premises, in particular at Prof Vicenzino and McPoil's homes. However, this data will be electronic in nature and accessed on a password-protected computer.

9c) What measures will be taken to ensure security of data during transit? (eg, if data is on hard-drive – protection by password/encryption/de-identification of data, etc).

Electronic data stored on a transportable hard drive (e.g. laptop computer) will be password-protected. It will also be anonymous in nature, as correlating consent forms will remain at the university site in hard copy.

9d) Will persons other than staff of the research team have access to the data?: NO

If YES, then please specify these persons, state why these persons have access, and what provisions are in place to ensure the confidentiality of data by these persons.

10) In what form will the data be <u>collected</u>:

Note: Tick the most appropriate box:

**(i) Identified** X	**(ii) Potentially Identifiable** ☐	**(iii) De-Identified** ☐ (ie, not able to be re-identified)

**11) In what form will the data be <u>stored and/or accessed</u>:** Note: Tick the most appropriate box:		
**(i) Identified** ☐	**(ii) Potentially Identifiable** X	**(iii) De-Identified** ☐ (ie, not able to be re-identified)

12) Give details of how feedback will be available to participants or informants:

A summary of study findings will be sent to all participants who provide their contact details for this purpose. A final paper outlining study design and findings will be written for the purposes of journal publication.

13) Does the project involve any of the following possibilities? Answer YES or NO. If YES, give details.

a) The trial or use of any medicine, drug, or other substance

1. Answer YES or NO. If YES, provide details:

No

2. Does this project require the submission of a Clinical Trial Notification/Clinical Trial Exemption (CTN/CTX) Form to the Therapeutic Goods Administration (TGA)? [Refer to the TGA website for further information]:

No

b) The trial of any device

1. Answer YES or NO. If YES, provide details:
Yes – the orthoses are used clinically by registered health care professionals, but they are being used for usually specified purposes.

2. Does this project require the submission of a Clinical Trial Notification/Clinical Trial Exemption (CTN/CTX) Form to the Therapeutic Goods Administration (TGA)? [Refer to the TGA website for further information]:

No

c) The trial of any intervention, therapy, or treatment (whether medical, behavioural, physical, or other)

Yes – the orthoses have been previously studied and are being used in accordance with standard use, purposes and application.

d) Any invasive procedures (eg, blood sampling)

No

e) Any diagnostic scans carried-out for the purposes of the project (including, *but not limited to*: MRI, NMR, CT/CAT, X-Rays, etc). No

1. If YES, please list.

2. Does your project involve the use of MRI? No

NOTE: If using MRI at a hospital site (i.e. a facility with emergency services available on site during testing),

you MUST have at least one staff who has current CPR certification and must have undertaken an emergency evacuation drill at least once a year.<br><br>If using MRI at non-hospital sites, (e.g. UQ St Lucia Campus), you MUST have 2 staff who both have current CPR certification and they must have undertaken an emergency evacuation drill at least once a year.<br><br>Does your project fulfil these mandatory conditions? N/a<br><br>If NO, outline reasons for submitting your application without these conditions in place.
3. Does your project involve exposure to ionising radiation? No<br><br>NOTE: If YES, the protocol MUST comply with the Queensland *Radiation Safety Act (1999)* and *Radiation Safety Regulation (2010)*. The legislation requires compliance with the Australian Radiation Protection and Nuclear Safety Agency's *Code of Practice for the Exposure of Humans to Ionising Radiation for research Purposes (ARPANSA 2005)* (http://www.arpansa.gov.au/pubs/rps/rps8.pdf) and you MUST consult with the University Radiation Protection Adviser before submission.<br><br>Does your project meet the guidelines of the Code of Practice? N/a<br><br>Has the project been reviewed by the University Radiation Protection Adviser before ethics submission? N/a

f) The possibility of physical stress/distress, or discomfort

1. to the participants:<br><br>As outlined previously, the purpose of this study is to establish potential improvements to comfort for those living with plantar heel pain. Neither members of the active control group nor of the placebo control group will conceivably have their discomfort increased as a result of participating.<br><br>2. to the researchers/data collectors:<br><br>No

g) The possibility of psychological/mental stress/distress, or discomfort

1. to the participants:<br><br>As stated previously, the potential for participants to become distressed upon discussing personal pain and function is negligible, as participants will be those whose plantar heel pain stems from a non-traumatic cause.<br><br>2. to the researchers/data collectors:<br><br>No

h) Deception of/or withholding information from, participant at **ANY** stage of the project

No

i) Access, by the investigators, to data held by a Commonwealth Department or Agency (Please also specify the number of records to be accessed)

No

j) Access, by the investigators, to data held by other bodies or people (Please also specify the number of records to be accessed)

No

k) Access to data (eg, medical records), by other bodies or people not the investigators.

No

l) Use of questionnaires, interviews, or focus groups with questions or topics which are sensitive, have potential to cause distress, or may reveal illegal activity

No

14) Please Indicate What You Think Is The Level Of Risk For Prospective Participants Against The Scale Below: *Tick the most appropriate box. (Refer to the UQ Guidelines)*

	**Extreme Risk**
	**High Risk**
	**Some Risk**
X	**Minimal Risk**
	**No Foreseeable Added Risk Above the Risks of Everyday Living**

15) Please provide details to assist the committee as to why you indicated the level of risk to prospective participants or informants in the question above (Question 14):

People participating in this study will have existing plantar heel pain for which they are not currently receiving treatment. The orthoses have been shown to reduce pain and disability in this patient population. The formed flip-flops have a similar foot bed as the orthoses and are likely to provide similar effects. Flat flip-flops are no different to those the participants would be wearing. For each individual participant, the allocated device will not be fitted if it is not comfortable for the participant to wear. This will minimise any adverse reaction to the devices.

16) How has the possibility of withdrawal from the project been addressed?:

Note: Ensure that details and effects of withdrawal without prejudice AT ANY TIME have been considered and explained. Refer to the NHMRC's *National Statement* section 2.2.19 – 2.2.20.

The researchers have accounted for a 10% attrition rate among participants when determining the desired sample size. Participants will be informed prior to recruitment that withdrawal at any stage is allowable without prejudice. Data collected from assessments will be re-identifiable (using the numbering system outlined above) in order to facilitate any requested withdrawals.

17) Please note that this section must be completed for funded research or the application will not be processed.

**17 a) Is this project receiving financial support to conduct the research?**	**YES** (circle)

17 b) If Yes, from what source(s)?

Vasyli International

17 c) Who will be administering the budget?

University of Queensland, Prof Bill Vicenzino

17 d) Please provide details of the budget distribution. (Or attach a copy of the budget statement.)

Total	$67,000.00	
Randomisation QCTC	$3,000.00	independent centre who will manage randomisation and concealled allocation
Physiotherapist	$22,500.00	$75 per session by 2 sessions per each participant to fit and to follow up once
Advertising	$36,000.00	3k allowance per advert for 12 months
Parking (travel) expenses	$3,000.00	2 visits at $10 per participant
Incidentals (copying, phone calls, including inter-investigator)	$2,000.00	
Hard drive for data storage	$500.00	

17 e) Provide details of any other "in kind" support for the project or direct or indirect payment to any investigator:

There is no direct payment to any investigator. The orthoses and flip flops will be supplied by Vasyli.

17 f) Please provide details of participant reimbursement for their involvement in the Project, if any:
Note: This could be cash payment, food vouchers, free services, or movie passes, etc.

Participants will be provided with orthoses or flip-flps to wear over the 12 weeks of the project. They will retain ownership of the shoes after the project is finished. A small amount ($20) will be paid to each participant for attending the lab for measurement at baseline and at 12 weeks.

18) In undertaking this research do any "conflict of interest" issues arise?
If YES, please provide details.
Note: Conflict of Interest may arise, for example, because a researcher, or someone close to the researcher, stands to benefit financially from the research or the carrying out of the project or because inconsistent or incompatible obligations exist.
Refer to section 5.4 of the NHMRC's *National Statement*:

No conflicts of interest are identified.

19) Is the project a multi-centre or site project?
If YES, provide the name of the principal ethics committee. Please provide copies of any conditions or requirements placed by other AHEC registered Human Ethics Committees:

**Note:** The Principal Ethics Committee is the Institutional Ethics Committee where the budget is to be administered.
As stated previously, the research team represent three participating universities: UQ (Prof Vicenzino); Regis University, Denver, USA (Prof McPoil); and University of Colorado, Denver, USA (Prof Mintken). Recruitment and data collection will be divided evenly between Brisbane (UQ School of Health and Rehabilitation Sciences site) and USA sites.<br><br>The principal ethics committee for this project is UQ MREC, as Prof Vicenzino is the Chief Investigator. Profs McPoil and Mintken will seek secondary ethics approval from their respective institutions. Any additional conditions or requirements imposed on the research team in the USA will be brought to the attention of UQ MREC, although this is not anticipated.

**20a) Some projects may involve permits from National Parks & Wildlife in relation to collection of data and Native Title issues. How have you addressed this issue?:** (Refer to the UQ Guidelines)	
N/a	
**20b) Does the project require biosafety clearance?**	**NO**<br>(circle)
